# Stuttering

**Published:** 1899-05

**Authors:** 


### Stuttering.

Liebmann (*Der Kinder-Arzt*) maintains that stuttering consists in a rushing together of the consonants, and consequently his method of treatment has for its object the teaching of the relative significance of the vowel and consonant sounds. The patient is made to speak sentences with prolonged vowels and short consonants, so that even at the first lesson many sentences are spoken easily and fluently. The physical effect is soon apparent, the patient regaining confidence in his ability to speak plainly, and the result is excellent in a very short time. This method can also be employed with young children. Stuttering may be caused by the infectious diseases, injury to the head, imitation of other children, or by heredity. Its more frequent occurrence in males than in females is to be explained by the greater motility of all the voluntary muscles in women than in men, the tongue included.—*Clinical Journal.*

The best disinfectant for a wound from which tetanus may be feared is peroxide of hydrogen, or, better still, hydrozone, which kills the bacillus.—*Med. Summary.*

